# Clinical and geriatric factors associated with fracture risk in older adults with bone metastases: A prospective observational study

**DOI:** 10.1007/s00520-026-10859-9

**Published:** 2026-06-04

**Authors:** İnci Güner, Serkan Akın, Nadir Yalçın

**Affiliations:** 1 Department of Clinical Pharmacy, Faculty of Pharmacy, Hacettepe University, 06100 Ankara, Türkiye; 2 Department of Medical Oncology, Hacettepe University Cancer Institute, Ankara, Türkiye

**Keywords:** Bone metastasis, Older adults, Fractures, Frailty, Malnutrition, Anticholinergic burden, Functional status

## Abstract

**Purpose:**

Bone metastases frequently lead to fractures, causing substantial morbidity, impaired quality of life, and poor prognosis in older adults with cancer. This study aimed to evaluate fracture risk by integrating geriatric parameters with clinical and oncological factors in older patients with bone metastases.

**Methods:**

This prospective observational study included 55 patients aged ≥ 65 years with newly diagnosed bone metastases who initiated monthly parenteral antiresorptive therapy. Baseline evaluation included standard oncological variables and validated geriatric tools (ADL, MUST, mFI-5, and ACB). Fracture occurrence was monitored over a six-month follow-up period. Univariate Cox regression analyses were performed to identify fracture-associated factors, followed by a theoretically driven multivariable Cox regression model. Correlation analyses were used to assess associations among significant variables.

**Results:**

Fractures occurred in 21 patients (38.2%). Univariate analyses showed that poorer performance status (ECOG ≥ 1; HR = 19.92), moderate functional impairment (ADL 3–5; HR = 8.39), high frailty (mFI-5 ≥ 2; HR = 9.83), osteolytic lesions (HR = 4.87), high anticholinergic burden (ACB ≥ 3; HR = 4.56), malnutrition risk (MUST ≥ 2; HR = 4.30), post-study falls (HR = 4.22), and lung metastases (HR = 2.97) were associated with increased fracture risk. Multivariable analysis identified ECOG score of 1 (aHR = 20.62) and high ACB score (aHR = 5.70) as independent predictors. Reduced functional independence and poorer performance status showed the strongest correlations with fracture occurrence.

**Conclusion:**

Fracture risk in older adults with bone metastases is driven predominantly by functional impairment, frailty, and medication-related vulnerability rather than tumor-related factors alone. Integrating geriatric assessment into routine oncology practice may improve fracture risk stratification and supportive care outcomes.

## Introduction

Population aging and continuous advances in oncological therapies have substantially increased both cancer incidence and survival among older adults. As a result, the burden of cancer in the geriatric population is projected to nearly double by 2040 [1, 2]. While prolonged survival represents a major therapeutic success, it has also led to a growing prevalence of long-term complications that directly affect functional status, independence, and quality of life. Among these, bone metastases have emerged as a critical clinical challenge, particularly in malignancies with a strong skeletal predilection such as prostate (85%), breast (70%), and lung cancers (40%) [3–5].

The increasing prevalence of bone metastases has been accompanied by a parallel rise in skeletal-related events, with fractures representing one of the most debilitating outcomes. In older adults with cancer, fracture incidence is reported to be up to 2.8-fold higher than in age-matched non-cancer populations [6]. These fractures are not merely structural complications but are closely linked to pain, immobility, loss of functional autonomy, increased hospitalization, and reduced quality of life, key endpoints within the scope of supportive cancer care.

Bone metastases compromise skeletal integrity through direct tumor-induced destruction; however, fracture risk extends beyond metastatic involvement alone. Cancer-directed therapies further impair bone health by disrupting bone remodeling, microarchitecture, and mechanical strength. Treatments such as chemotherapy, hormone deprivation therapy, and oophorectomy induce hypogonadal states that accelerate bone loss, while emerging therapies, including immune checkpoint inhibitors, may promote bone resorption via T-cell–mediated cytokine activity. Consequently, patients may sustain fractures following low-energy trauma, even when bone mineral density (BMD) values do not meet osteoporotic thresholds [7, 8]. These mechanisms underscore the limitations of traditional bone health assessments when applied in isolation to oncology populations.

Importantly, in older adults, fracture risk is further amplified by geriatric syndromes that are central to supportive care [9–11]. Frailty, functional impairment, malnutrition, polypharmacy, and increased fall risk synergistically contribute to skeletal vulnerability and may precipitate pathological fractures at metastatic sites, even in the absence of significant trauma [12]. Fracture occurrence in this population is associated with poorer clinical outcomes, diminished quality of life, and substantially increased healthcare utilization and costs [13, 14]. Given these challenges, preserving skeletal integrity is critical for maintaining mobility, functional autonomy, and overall quality of life in patients with bone metastases [3].

Despite the clinical relevance of these factors, fracture risk assessment in patients with bone metastases remains largely focused on tumor burden and radiological features, with limited integration of geriatric parameters. There is a critical need for comprehensive, clinically applicable risk stratification models that incorporate both oncological and geriatric domains to inform preventive strategies and individualized supportive interventions. Accordingly, the present study aimed to evaluate the predictive value of geriatric risk factors alongside clinical and oncological variables to provide an integrated fracture risk assessment in older adults with bone metastatic disease, thereby supporting more effective and patient-centered supportive care strategies.

## Methods

### Study design and participants

This prospective, observational, single-center study was conducted between January 1, 2025, and November 1, 2025, at the Day Treatment Unit of Hacettepe University Cancer Institute, a comprehensive cancer center in Ankara, Türkiye. The study population consisted of older adults with solid tumors and newly diagnosed bone metastases. Eligibility criteria included: (1) a confirmed new diagnosis of bone metastasis; (2) initiation of monthly parenteral antiresorptive therapy; (3) age ≥ 65 years; and (4) provision of written informed consent. Exclusion criteria were: (1) the presence of psychological or cognitive disorders impairing effective communication; (2) an Eastern Cooperative Oncology Group (ECOG) performance status of 3–4; and (3) a prior history of antiresorptive therapy.

### Data collection and clinical assessment

Data were collected from electronic health records and supplemented by structured, face-to-face patient assessments. Baseline demographic and anthropometric characteristics recorded at study enrollment included age, sex, weight, height, body mass index (BMI), smoking status, alcohol consumption, and physical activity level, categorized as inactive/sedentary or active/regular exercise.

Clinical and oncological variables comprised ECOG performance status, primary tumor histology, presence of visceral metastases, and radiological characteristics of bone involvement, classified as osteolytic, osteoblastic, or mixed lesions. Details of systemic anticancer treatments (chemotherapy, targeted therapy, immunotherapy, combination regimens, or palliative treatment) and the type of administered antiresorptive agent were documented.

Medication-related parameters included the total number of daily medications, polypharmacy status, comorbid conditions, and Charlson Comorbidity Index (CCI) scores. In addition, a history of falls within the year preceding study enrollment, bone mineral density (BMD) measurements of the lumbar spine and femoral neck, and baseline nutritional status (regular diet or use of oral nutritional supplements) were recorded.

### Comprehensive geriatric assessment

A comprehensive geriatric assessment was performed at baseline using validated instruments, including the Katz Index of Activities of Daily Living (ADL), the Malnutrition Universal Screening Tool (MUST), the Modified Frailty Index-5 (mFI-5), and the Anticholinergic Cognitive Burden (ACB) scale. During follow-up, the total number of administered antiresorptive doses was documented, and fall events occurring throughout the 6-month observation period were systematically recorded as present or absent. The functional independence of the participants was evaluated using the Katz Index of Independence in ADL. Among instruments used to assess basic ADLs, the Katz ADL is the most well-known tool in clinical practice and the most widely utilized in clinical studies. Developed by Katz et al. [15, 16], the index measures performance in six self-care tasks: bathing, dressing, toileting, transferring, maintaining continence, and feeding. The six-item Katz ADL is brief and can be administered via interview. In this study, functional status was categorized based on the total score: a score of 6 indicates the patient is independent, 3–5 indicates moderate impairment, and 0–2 indicates that the patient is very dependent.

Nutritional risk was assessed using the MUST [17], in accordance with the recommendations of the European Society for Parenteral and Enteral Nutrition and the British Association for Parenteral and Enteral Nutrition for the identification of malnutrition and obesity risk in adults. The MUST score is derived from three components: BMI, unintentional weight loss during the preceding 3–6 months, and the presence of an acute disease effect. Based on the total score, patients were classified as low risk (score 0), medium risk (score 1), or high risk (score ≥ 2).

Frailty status was evaluated using the modified 5-item Frailty Index (mFI-5), as described by Subramaniam et al. [18]. The index is calculated based on the presence of five predefined clinical variables: diabetes mellitus, congestive heart failure, hypertension requiring pharmacological treatment, chronic obstructive pulmonary disease, and non-independent functional status. Each variable was assigned one point when present, yielding a total score ranging from 0 to 5. Based on the cumulative score, patients were categorized as non-frail (score 0), mildly frail (score 1), or having moderate-to-severe frailty (score ≥ 2) [19].

Cumulative anticholinergic burden was assessed using the Anticholinergic Burden Calculator (www.acbcalc.com), a digital tool that integrates the Anticholinergic Cognitive Burden (ACB) scale and the German Anticholinergic Burden Scale (GABS) [20, 21]. This tool quantifies the cumulative anticholinergic effects of medications on cognitive and physical function in older adults by assigning scores based on anticholinergic potency: no activity (score 0), possible activity (score 1), and definite activity (score 2 or 3). The total anticholinergic burden score was used to classify patients into three categories: low burden (score 0), moderate burden (score 1), and high burden (score ≥ 3).

### Outcome assessment

Fracture outcomes were ascertained through a systematic review of radiographic reports within the electronic medical record system. Only fractures that were radiologically confirmed and documented at the study institution were included. Self-reported fractures without radiographic verification were excluded from the analysis to ensure diagnostic accuracy.

The relatively small sample size reflects the application of strict inclusion criteria, which limited enrollment to patients with newly diagnosed bone metastases initiating antiresorptive therapy. This approach was intentionally adopted to reduce confounding related to prior bone-targeted treatments and to ensure a more homogeneous study population.

All patients received standardized nutritional recommendations during the six-month antiresorptive treatment period, including a calcium- and protein-rich diet and routine supplementation with calcium and vitamin D₃. Patients identified with vitamin D deficiency were managed in accordance with established clinical practice guidelines.

### Statistical analysis

Statistical analyses were performed using SPSS software (version 26.0; IBM Corp., Armonk, NY, USA). The distribution of continuous variables was assessed using both visual inspection (histograms and Q–Q plots) and formal normality tests (Kolmogorov–Smirnov and Shapiro–Wilk tests). Categorical variables were expressed as frequencies and percentages.

Comparisons between patients with and without bone fractures were conducted using the Pearson chi-square (χ^2^) test or Fisher’s exact test for categorical variables, as appropriate. Continuous variables were compared using the Student’s t-test for normally distributed data or the Mann–Whitney U test for non-normally distributed data. Associations between variables were evaluated using Spearman’s rank correlation coefficient (rₛ).

The effects of potential risk factors on fracture occurrence were examined using univariate Cox proportional hazards regression analysis. Variables that were found to be statistically significant in the univariate analysis (*p* < 0.05) were subsequently included in a multivariate Cox proportional hazards regression model using a backward stepwise selection method to identify independent risk factors for bone fractures. Results were expressed as hazard ratios (HRs) and adjusted hazard ratios (aHRs) with corresponding 95% confidence intervals (CIs). Variables included in the multivariate Cox regression model were selected based on clinical relevance and statistical significance in univariate analysis, while avoiding collinearity among closely related geriatric parameters.

In addition to Cox proportional hazards regression, time-to-event analyses were performed using the Kaplan–Meier method to evaluate fracture-free survival during the six-month follow-up period. Time to fracture was defined as the interval from the initiation of antiresorptive therapy (baseline visit) to the occurrence of the first radiologically confirmed bone fracture. Patients who did not experience a fracture during follow-up were censored at the time of last clinical assessment. Patients who died before completing the follow-up period without developing a fracture were censored at the time of death. Kaplan–Meier curves were generated for variables that were significant in univariate analysis, and differences between survival curves were assessed using the log-rank test. A two-sided *p* value < 0.05 was considered statistically significant for all analyses.

## Results

### Patient characteristics and fracture incidence

A total of 55 patients were included in the study. During the six-month follow-up period, bone fractures occurred in 21 patients (38.2%), whereas 34 patients (61.8%) did not experience any fractures. Of the patients with fractures, 9 (42.9%) were diagnosed following clinical presentations of refractory severe pain and loss of mobility, while 12 (57.1%) were identified incidentally during routine periodic restaging scans. Radiological evaluation revealed that compression (collapse) fractures were present in 19 patients (90.4%), while non-compression fractures were observed in 10 patients (47.6%). Regarding the coexistence of fracture types, 11 patients presented with isolated compression fractures and 2 patients with isolated non-compression fractures, whereas 8 patients exhibited both fracture patterns simultaneously. As detailed in Table 1, compression fractures were primarily localized in the junctional spine (*n* = 16) and thoracic spine (*n* = 9) regions. Non-compression fractures were distributed across the ribs/chest wall (*n* = 6), hip/acetabulum (*n* = 2), long bones (*n* = 2), and spinal processes (*n* = 1).

**Table 1:** Distribution and characteristics of fractures (*n* = 21)

Fracture Category/Location	Patients (*n*, %)
I. Compression (Collapse) Fractures	19 (90.4)
*Junctional Spine*	16 (76.1)
*Thoracic Spine*	9 (42.8)
II. Non-compression Fractures	10 (47.6)
*Ribs/Chest Wall*	6 (28.5)
*Hip/Acetabulum*	2 (9.5)
*Long Bone*	2 (9.5)
*Spinal Processes*	1 (4.7)
III. Co-existence of Fracture Types	
*Isolated Compression Fractures*	11 (52.3)
*Isolated Non-compression Fractures*	2 (9.5)
*Combined (Compression* + *Non-compression)*	8 (38)

The study population was stratified into two groups based on fracture occurrence: bone fracture–positive and bone fracture–negative. A comparative analysis of demographic and clinical characteristics between these groups is summarized in Table 2.

**Table 2:** Demographic and clinical characteristics of the patients

Variables	Total (*n* = 55)	Bone fracture (+) (*n* = 21, 38.2%)	Bone fracture (-) (*n* = 34, 61.8%)	*p*-value
Gender, *n* (%)				
*Female*	24 (43.6)	9 (42.9)	15 (44.1)	0.927
*Male*	31 (56.4)	11 (57.1)	20 (55.9)	
Age (years), mean (SD)	72.71 (5.91)	71.10 (4.08)	73.71 (6.65)	0.270
Weight (kg), mean (SD)	67.55 (10.97)	65.52 (9.32)	68.89 (11.84)	0.287
Height (cm), mean (SD)	164.42 (8.16)	164.33 (8.49)	164.47 (8.07)	0.952
BMI (kg/m^2^), mean (SD)	24.99 (3.79)	24.35 (3.73)	25.38 (3.83)	0.275
Smoking, *n* (%)	31 (56.4)	12 (57.1)	19 (55.9)	0.927
Alcohol, *n* (%)	11 (20)	4 (19)	7 (20.6)	0.588
Physical activity status, *n* (%)				
*Inactive (sedentary)*	44 (80)	20 (95.2)	24 (70.6)	**0.025**
*Active (regular walking/exercise)*	11 (20)	1 (4.8)	10 (29.4)	
Performance status (ECOG), *n* (%)				
*0*	43 (78.2)	11 (52.4)	32 (94.1)	
*1*	7 (12.7)	7 (33.3)	-	** < 0.001**
*2*	5 (9.1)	3 (14.3)	2 (5.9)	
Histology, *n* (%)				
*Lung*	16 (29.1)	6 (28.6)	10 (29.4)	
*Prostate*	11 (20)	6 (28.6)	5 (14.7)	0.652
*Breast*	6 (10.9)	2 (9.5)	4 (11.8)	
*Other*	22 (40)	7 (33.3)	15 (44.1)	
Visceral metastasis, *n* (%)	33 (60)	13 (61.9)	20 (58.8)	0.821
*Liver*	16 (29.1)	7 (33.3)	9 (26.5)	0.586
*Lung*	15 (27.3)	9 (42.9)	6 (17.6)	**0.041**
*Brain*	8 (14.5)	5 (23.8)	3 (8.8)	0.236
*Spleen*	7 (12.7)	2 (9.5)	5 (14.7)	0.696
Bone lesion type, *n* (%)				
*Osteosclerotic*	22 (40)	5 (23.8)	17 (50)	
*Osteolytic*	13 (23.6)	8 (38.1)	5 (14.7)	**0.046**
*Mixed*	13 (23.6)	7 (33.3)	6 (17.6)	
*Unknown*	7 (12.7)	1 (4.8)	6 (17.6)	
Systemic anticancer regimens, *n* (%)				
*Chemotherapy*	19 (34.5)	7 (33.3)	12 (35.3)	
*Targeted therapy*	12 (21.8)	3 (14.3)	9 (26.5)	
*Immunotherapy*	3 (5.5)	3 (14.3)	-	0.267
*Combination*	11 (20)	4 (19)	7 (20.6)	
*Palliative treatment*	10 (18.2)	4 (19)	6 (17.6)	
Antiresorptive therapy, *n* (%)				
*Zoledronic acid*	29 (52.7)	11 (52.4)	18 (52.9)	0.968
*Denosumab*	26 (47.3)	10 (47.6)	16 (47.1)	
Number of antiresorptive, mean (SD)	4.51 (1.54)	4.62 (1.54)	4.33 (1.56)	0.508
Total number of daily drugs, mean (SD)	8.6 (2.93)	8.33 (2.08)	8.76 (3.37)	0.600
Polypharmacy (≥ 5), *n* (%)	51 (92.7)	21 (100)	30 (88.2)	0.286
Comorbidities, mean (SD)	1.91 (1.54)	2.12 (1.59)	1.57 (1.43)	0.212
Diagnosis, *n* (%)				
*Hypertension*	31 (56.4)	14 (66.7)	17 (50)	0.226
*Coronary artery disease*	18 (32.7)	4 (19)	14 (41.2)	0.089
*Diabetes mellitus*	13 (23.6)	5 (23.8)	8 (23.5)	0.614
*Benign prostatic hyperplasia*	11 (20)	5 (23.8)	6 (17.6)	0.412
*Hyperlipidemia*	9 (16.4)	1 (4.8)	8 (23.5)	0.068
*Hypothyroidism*	8 (14.5)	3 (14.3)	5 (14.7)	0.644
*Atrial fibrillation*	7 (12.7)	2 (9.5)	5 (14.7)	0.454
*Chronic kidney disease*	6 (10.9)	2 (9.5)	4 (11.8)	0.584
*Chronic obstructive pulmonary disease*	3 (5.5)	-	3 (8.8)	-
*Congestive heart failure*	2 (3.6)	1 (4.8)	1 (2.9)	0.622
*Gastroesophageal reflux disease*	2 (3.6)	-	2 (5.9)	-
*Liver failure*	2 (3.6)	1 (4.8)	1 (2.9)	0.599
Charlson comorbidity index, mean (SD)	10.05 (1.37)	10.26 (1.31)	9.71 (1.42)	0.163
Pre-study falls (1 year), *n* (%)	8 (14.5)	3 (14.3)	5 (14.7)	1.000
Post-study falls (6 months), *n* (%)	11 (20)	9 (42.9)	2 (5.9)	**0.001**
Bone mineral density score, lumbar *n* (%)				
≥ *−1.0 (normal)*	26 (47.3)	12 (57.1)	14 (41.2)	
*−1.0 to −2.5 (osteopenia)*	11 (20)	1 (4.8)	10 (29.4)	0.104
≤ *−2.5 (osteoporosis)*	7 (12.7)	3 (14.3)	4 (11.8)	
*Unknown*	11 (20)	5 (23.8)	6 (17.6)	
Bone mineral density score, femur, *n* (%)				
≥ *−1.0 (normal)*	17 (30.9)	8 (38.1)	9 (26.5)	
*−1.0 to −2.5 (osteopenia)*	15 (27.3)	4 (19)	11 (32.4)	0.491
≤ *−2.5 (osteoporosis)*	11 (20)	4 (19)	7 (20.6)	
*Unknown*	12 (21.8)	5 (23.8)	7 (20.6)	
Nutritional status, *n* (%)				
*Normal diet*	35 (63.6)	11 (52.4)	24 (70.6)	0.173
*Oral nutritional supplements*	20 (36.4)	10 (47.6)	10 (29.4)	
Modified frailty index-5 score, *n* (%)				
*0 (non-frail)*	12 (21.8)	1 (4.8)	11 (32.4)	
*1 (mild frailty)*	23 (41.8)	9 (42.9)	14 (41.2)	**0.031**
≥ *2 (moderate-to-severe frailty)*	20 (36.4)	11 (52.4)	9 (26.5)	
Activities of daily living score, *n* (%)				
*6 (independent)*	35 (63.6)	6 (28.6)	29 (85.3)	
*3–5 (moderate impairment)*	17 (30.9)	14 (66.7)	3 (8.8)	** < 0.001**
*0–2 (very dependent)*	3 (5.5)	1 (4.8)	2 (5.9)	
Malnutrition universal screening tool score, *n* (%)				
*0 (low risk)*	18 (32.7)	3 (14.3)	15 (44.1)	
*1 (medium risk)*	12 (21.8)	4 (19)	8 (23.5)	**0.030**
≥ *2 (high risk)*	25 (45.5)	14 (66.7)	11 (32.4)	
Anticholinergic cognitive burden score, *n* (%)				
*0 (low risk)*	14 (25.5)	3 (14.3)	11 (32.4)	
*1–2 (medium risk)*	26 (47.3)	8 (38.1)	18 (52.9)	**0.024**
≥ *3 (high risk)*	15 (27.3)	10 (47.6)	5 (14.7)	

Baseline demographic characteristics, including sex, age, weight, height, BMI, and smoking and alcohol consumption, were comparable between the two groups (all *p* > 0.05), indicating a homogeneous distribution of these parameters. In contrast, physical activity status differed significantly, with lower levels of regular physical activity observed in the fracture group (*p* = 0.025).

No statistically significant differences were observed between groups with respect to primary tumor histology, systemic anticancer treatment regimens, antiresorptive therapy (use and cumulative dose), baseline nutritional status, total number of daily medications, polypharmacy (≥ 5 medications), comorbidity burden, CCI scores, history of falls within the year preceding enrollment, or BMD measurements of the lumbar spine and femur (all *p* > 0.05).

Significant between-group differences were identified in radiological and metastatic characteristics. Osteolytic bone lesions were more frequently observed in the fracture group compared with the non-fracture group (38.1% vs. 14.7%, *p* = 0.046). Similarly, the presence of lung metastases was significantly higher among patients who developed fractures (42.9% vs. 17.6%, *p* = 0.041).

With respect to functional and geriatric parameters, patients in the fracture group demonstrated significantly poorer ECOG performance status (*p* < 0.001) and greater functional dependency as assessed by ADL scores (*p* < 0.001). Higher frailty levels according to the modified 5-item Frailty Index (mFI-5) (*p* = 0.031), increased risk of malnutrition based on the MUST (*p* = 0.030), and greater ACB (*p* = 0.024) were also significantly more prevalent in patients with fractures. In addition, falls occurring during the follow-up period were markedly more frequent in the fracture group than in the non-fracture group (*p* = 0.001).

### Predictors of fracture risk

The univariate analysis of factors associated with bone fracture risk is summarized in Table 3. The strongest predictors of fracture occurrence were poor performance status (ECOG 1: HR = 19.925, 95% CI: 6.392–62.108, *p* < 0.001), elevated frailty burden (mFI-5 ≥ 2: HR = 9.838, 95% CI: 1.266–76.430, *p* = 0.029), and moderate functional impairment (ADL score 3–5: HR = 8.399, 95% CI: 3.172–22.244, *p* < 0.001).

**Table 3:** Risk factors for bone fracture

Variables	Univariate analysis Hazard ratio (%95 CI)	*p*-value	Multivariate analysis Adjusted hazard ratio (%95 CI)	*p*-value
Physical activity status				
*Active (regular walking/exercise)*	1.00 (reference)			
*Inactive (sedentary)*	7.184 (0.961–53.684)	0.055		
Performance status (ECOG)				
*0*	1.00 (reference)			
*1*	19.925 (6.392–62.108)	** < 0.001**	20.619 (5.823–73.005)	** < 0.001**
*2*	3.538 (0.972–12.880)	0.055	7.618 (1.849–31.392)	0.005
Lung metastasis				
*No*	1.00 (reference)			
*Yes*	2.974 (1.245–7.103)	**0.014**		
Bone lesion type				
*Osteosclerotic*	1.00 (reference)			
*Osteolytic*	4.877 (1.557–15.272)	**0.007**		
*Mixed*	3.525 (1.115–11.143)	**0.032**		
Post-study falls (6 months)				
*No*	1.00 (reference)			
*Yes*	4.221 (1.764–10.104)	**0.001**		
Modified frailty index-5 score				
*0 (non-frail)*	1.00 (reference)			
*1 (mild frailty)*	5.982 (0.757–47.256)	0.090		
≥ *2 (moderate-to-severe frailty)*	9.838 (1.266–76.430)	**0.029**		
Activities of daily living score				
*6 (independent)*	1.00 (reference)			
*3–5 (moderate impairment)*	8.399 (3.172–22.244)	** < 0.001**		
*0–2 (very dependent)*	2.596 (0.311–21.665)	0.378		
Malnutrition universal screening tool score				
*0 (low risk)*	1.00 (reference)			
*1 (medium risk)*	1.991 (0.445–8.900)	0.367		
≥ *2 (high risk)*	4.305 (1.234–15.013)	**0.022**		
Anticholinergic cognitive burden score				
*0 (low risk)*	1.00 (reference)			
*1–2 (medium risk)*	1.609 (0.427–6.070)	0.482	1.897 (0.499–7.212)	0.348
≥ *3 (high risk)*	4.563 (1.250–16.656)	**0.022**	5.695 (1.430–22.678)	**0.014**

Additional variables associated with a statistically significant increase in fracture risk included osteolytic bone lesions (HR = 4.877, *p* = 0.007), high anticholinergic burden (ACB ≥ 3: HR = 4.563, *p* = 0.022), high malnutrition risk (MUST ≥ 2: HR = 4.305, *p* = 0.022), and the occurrence of falls during the follow-up period (HR = 4.221, *p* = 0.001). The presence of lung metastasis was also identified as a significant clinical predictor, conferring nearly a threefold increase in fracture risk (HR = 2.974, *p* = 0.014).

Several variables did not reach statistical significance in the univariate model. Physical activity status and ECOG performance status of 2 demonstrated a trend toward increased fracture risk but remained at the trend towards of statistical significance (*p* = 0.055 for both). Similarly, intermediate-risk categories of mFI-5 (*p* = 0.090), MUST (*p* = 0.367), and ACB (*p* = 0.482), as well as the highest level of functional dependency according to ADL scores (*p* = 0.378), were not significantly associated with fracture risk.

The nine risk factors found to be significant in the univariate analysis (Physical activity status, ECOG, lung metastasis, bone lesion type, post-study falls, mFI-5, ADL, MUST, and ACB score) were included in a Backward Stepwise Cox Regression model. To ensure model stability, collinearity diagnostics were performed prior to the multivariate analysis; the Variance Inflation Factor (VIF) for all variables ranged from 1.18 to 3.17, indicating no significant multicollinearity. In the final step, ECOG performance status (*p* < 0.001) and ACB score (*p* = 0.014) were identified as the strongest independent risk factors for fracture development. Specifically, patients with an ECOG score of 1 demonstrated a 20.6-fold increase in fracture risk (adjusted Hazard Ratio [aHR]: 20.619, 95% CI: 5.823–73.005), while those with a high ACB score (≥ 3) exhibited a 5.7-fold increased risk (aHR: 5.695, 95% CI: 1.430–22.678) compared to the reference groups.

### Correlation analysis

Variables that reached statistical significance in the univariate analysis (*p* < 0.05) were included in Spearman’s correlation analysis to evaluate the strength and direction of their associations (Table 4). Bone fracture status demonstrated significant correlations with all variables included in the correlation matrix. The strongest associations were observed with ADL scores (rₛ =  − 0.531, *p* < 0.01) and ECOG performance status (rₛ = 0.464, *p* < 0.01), indicating that lower functional independence and poorer performance status were moderately to strongly associated with fracture occurrence.

**Table 4:** Spearman Correlation Matrix of variables significant in univariate analysis

Variables (r_s_)	1	2	3	4	5	6	7	8	9	10
1. Bone fracture	1									
2. ECOG	0.464**	1								
3. ADL	−0.531**	−0.736**	1							
4. MUST	0.357**	0.444**	−0.511**	1						
5. mFI-5	0.341*	0.375**	−0.453**	0.239	1					
6. ACB	0.342*	0.165	−0.243	0.166	0.396**	1				
7. Post-study falls	0.449**	0.405**	−0.366**	0.245	0.207	0.113	1			
8. Lung metastasis	0.275*	0.257	−0.366**	0.284*	0.164	0.097	0.102	1		
9. Bone lesion type	0.307*	0.245	−0.307*	0.212	0.270	0.087	0.229	0.098	1	
10. Physical activity	−0.299*	−0.262	0.373**	−0.122	−0.218	−0.264	−0.250	−0.204	−0.286*	1

*ECOG* Eastern Cooperative Oncology Group performance status; *ADL* Activities of Daily Living; *MUST* Malnutrition Universal Screening Tool; *mFI-5* Modified 5-item Frailty Index; *ACB* Anticholinergic Cognitive Burden

* *p* < 0.05, ** *p* < 0.01

Moderate but statistically significant correlations were also identified between bone fracture and post-study falls (rₛ = 0.449, *p* < 0.01), MUST scores (rₛ = 0.357, *p* < 0.01), and mFI-5 scores (rₛ = 0.341, *p* < 0.05). Physical activity level was negatively correlated with fracture occurrence (rₛ =  − 0.299, *p* < 0.05), suggesting a protective association; however, this variable remained at the trend towards of statistical significance in the univariate analysis (*p* = 0.055).

Strong interrelationships were observed among functional and geriatric parameters. ECOG performance status showed a strong negative correlation with ADL scores (rₛ =  − 0.736, *p* < 0.01), reflecting the close association between overall performance and functional independence. ADL scores were also significantly and negatively correlated with MUST (rₛ =  − 0.511, *p* < 0.01) and mFI-5 (rₛ =  − 0.453, *p* < 0.01). In parallel, ECOG performance status demonstrated positive correlations with MUST (rₛ = 0.444, *p* < 0.01) and post-study falls (rₛ = 0.405, *p* < 0.01). Additionally, a significant positive correlation was observed between mFI-5 and ACB scores (rₛ = 0.396, *p* < 0.01), while lung metastasis showed a significant negative correlation with ADL scores (rₛ =  − 0.366, *p* < 0.01).

### Anticholinergic medication profile

Consistent with the findings of the univariate and correlation analyses, which demonstrated significantly higher ACB scores in the fracture group, the mean number of anticholinergic medications per patient was also significantly greater in this cohort (*p* = 0.001). Overall, 16 distinct medications with anticholinergic properties were identified, comprising 44 prescriptions in the fracture group and 33 prescriptions in the non-fracture group. According to the ACB scoring system, 13 medications were assigned 1 point, one medication was assigned 2 points, and two medications were assigned 3 points.

In both groups, the most frequently prescribed anticholinergic agents were pantoprazole (ACB score 1) and tramadol (ACB score 2). Notably, the prevalence of tramadol use (42.9% vs. 17.6%; *p* = 0.041) and prednisolone use (23.8% vs. 2.9%; *p* = 0.026) was significantly higher among patients who developed fractures compared with those who did not (Table 5).

**Table 5:** Daily anticholinergic drug use in patients with and without bone fractures

Variables	Bone fracture (+) (*n* = 21, 38.2%)	Bone fracture (-) (*n* = 34, 61.8%)	Total (*n* = 55, 61.8%)	*p*-value
Drugs, (*n* %)				
Pantoprazole^†^	9 (42.9)	7 (20.6)	16 (29.1)	0.077
Tramadol^‡^	9 (42.9)	6 (17.6)	15 (27.3)	**0.041**
Metformin^†^	4 (19.0)	5 (14.7)	9 (16.4)	0.719
Fentanyl^†^	4 (19.0)	2 (5.9)	6 (10.9)	0.188
Prednisolone^†^	5 (23.8)	1 (2.9)	6 (10.9)	**0.026**
Morphine^†^	4 (19.0)	1 (2.9)	5 (9.1)	0.064
Lansoprazole^†^	-	4 (11.8)	4 (7.3)	-
Sertraline^†^	2 (9.5)	1 (2.9)	3 (5.5)	0.551
Celecoxib^†^	3 (14.3)	-	3 (5.5)	-
Dexamethasone^†^	2 (9.5)	-	2 (3.6)	-
Tolterodine^§^	1 (4.8)	1 (2.9)	2 (3.6)	1.000
Digoxin^†^	1 (4.8)	1 (2.9)	2 (3.6)	1.000
Cetirizine^†^	-	1 (2.9)	1 (1.8)	-
Alverine^†^	-	1 (2.9)	1 (1.8)	-
Fluoxetine^†^	-	1 (2.9)	1 (1.8)	-
Amitriptyline^§^	-	1 (2.9)	1 (1.8)	-
Anticholinergic drugs/patient, mean (SD)	2.10 (1.37)	0.97 (0.90)	1.4 (1.23)	**0.001**

According to the Anticholinergic Cognitive Burden Scale; ^†^1 point, ^‡^2 points, ^§^3 points

### Survival analysis

Kaplan–Meier survival analysis demonstrated significant differences in fracture-free survival according to key geriatric and clinical parameters. Patients with impaired functional status (ADL score 3–5) exhibited a significantly shorter time to fracture compared with functionally independent patients (log-rank *p* < 0.001). Similarly, patients with poorer performance status (ECOG ≥ 1) experienced earlier fracture events than those with ECOG 0 (log-rank *p* < 0.001).

A significantly reduced fracture-free survival was also observed in patients with moderate-to-severe frailty (mFI-5 ≥ 2; log-rank *p* = 0.028), high malnutrition risk (MUST ≥ 2; log-rank *p* = 0.021), and high anticholinergic burden (ACB ≥ 3; log-rank *p* = 0.019).

**Fig. 1:**
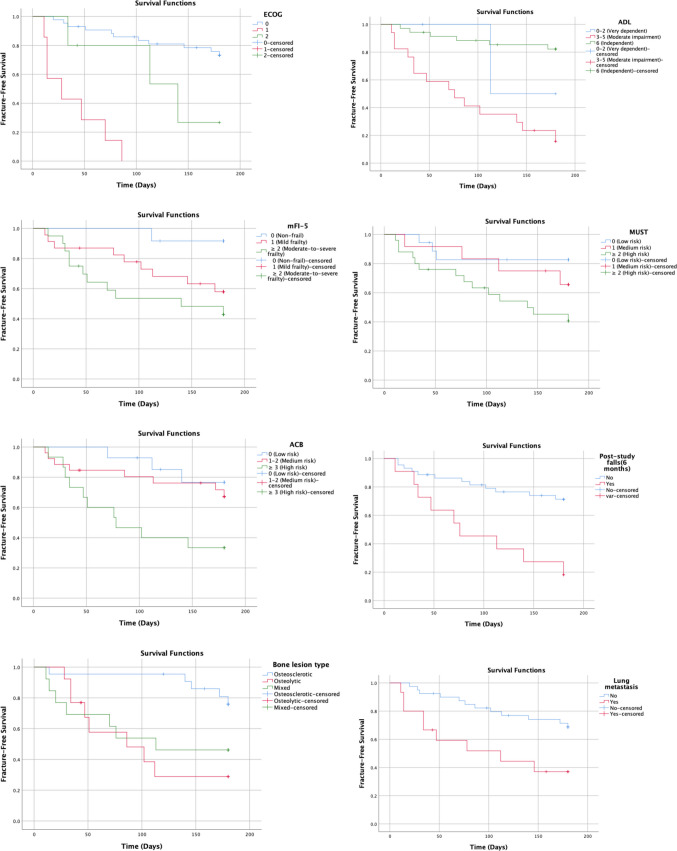
Kaplan–Meier curves illustrating fracture-free survival during the follow-up period. The analysis depicts time to the first skeletal-related event (bone fracture) and demonstrates the impact of selected clinical and geriatric variables on fracture occurrence over time. Patients who did not experience a fracture during follow-up were censored at the time of last assessment

Furthermore, patients who experienced falls during the follow-up period had a markedly shorter time to fracture compared with those without falls (log-rank *p* = 0.001). The presence of osteolytic bone lesions and lung metastasis was also associated with significantly earlier fracture occurrence (log-rank *p* = 0.007 and *p* = 0.014, respectively). During follow-up, five patients died without experiencing a fracture and were censored at the time of death in the Kaplan–Meier analysis (Fig. 1).

## Discussion

To our knowledge, this is among the first prospective studies to investigate the development of fractures in older adults with bone metastases, incorporating geriatric parameters. Although the literature indicates that the risk of pathological fractures varies widely between 17 and 50% depending on the primary tumor histology and anatomical distribution [22], our observed rate of 38% falls squarely within this reported range. This finding not only reinforces the validity of our results but also highlights that the risk of fracture remains substantial in the older adult population.

In our study, although almost all patients with bone metastases experienced varying degrees of pain, a significant portion of those with fractures did not report any new symptoms, and these fractures were identified during routine restaging scans. Conversely, 9 patients (42.9%) presented with refractory severe pain and loss of mobility, which prompted the radiological imaging that led to their diagnosis. The high proportion of patients not reporting symptoms may stem from a lack of awareness regarding symptom reporting. Early detection is vital in this group to prevent pain and functional decline from intensifying and rapidly progressing toward increased morbidity and mortality. Findings facilitated by regular imaging and geriatric screenings enable the timely initiation of interventions, such as radiotherapy or surgery, thereby ensuring skeletal stabilization and improving the patient’s quality of life.

Regarding the anatomical distribution, a high degree of co-location was observed between bone metastases and fracture sites, particularly within the axial skeleton where compression fractures predominated. This alignment underscores the predominantly pathological nature of these events, as the disruption of bone mineral homeostasis and impaired remodeling at metastatic sites severely compromise the mechanical load-bearing capacity of the vertebrae. However, our findings suggest that fracture occurrence in older adults is not solely a function of tumor burden; the significant correlation between falls and fracture development indicates that systemic geriatric vulnerability acts as a catalyst for mechanical failure at metastatic sites.

This research investigated the multifaceted factors contributing to bone fracture risk in older patients with metastatic bone disease. The most striking finding of our study is that in older adults with bone metastases, the geriatric risk factors including performance status, functional dependence, frailty, malnutrition, and anticholinergic drug burden emerge as significant predictors of fracture risk.

In the univariate analysis results of our study, it was determined that ECOG 1 performance status was associated with an approximately 20-fold increase in fracture risk, and ADL moderate impairment status was associated with an eightfold increase. Similarly, a post-study 6-month history of falls was associated with a fourfold increase in fracture risk, reinforcing the clinical consensus that evaluating fall risk factors is mandatory, as they significantly dictate fracture likelihood in older patients and are often overlooked during routine oncologic evaluations [11]. Furthermore, our multivariate analysis confirmed that ECOG performance status remains the strongest factor independently associated with fracture risk; specifically, patients with an ECOG score of 1 demonstrated a 20.6-fold increased risk, highlighting that even a slight decline in functional capacity significantly escalates the probability of skeletal events. Despite a trend toward increased risk, the ECOG 2 and ADL very dependent groups failed to reach statistical significance; a finding that diverges from the theoretical expectation that higher disability scores would confer a proportionately greater risk. This observation is likely attributable to the limited number of patients within these categories, which weakens statistical power. Furthermore, the limited mobilization of patients with such high dependency and low performance scores may have reduced their exposure to factors like mechanical loading or falls, which are triggers in the fracture mechanism. Our findings support this mechanical-risk hypothesis through the recorded physical activity levels. Specifically, the low incidence of fractures observed in the active (regular walking/exercise) group suggests that maintaining a degree of regular physical activity may serve as a marker of a more resilient skeletal system and better muscle strength, which contributes to bone stability. In contrast, patients with ECOG 1 appear to occupy a high-risk mobility zone; they remain active enough to encounter mechanical stresses but lack the skeletal integrity to withstand them. Meanwhile, the lower hazard ratio in ECOG 2 patients, compared to ECOG 1, reflects a paradoxical protective effect of severe immobility. In these frail individuals, the dramatic reduction in weight-bearing activities and environmental triggers likely prevents the acute mechanical events required to provoke a fracture. Our findings suggest a potential non-linear association between functional status and fracture risk. Patients with moderate impairment are mobile enough to encounter trauma but frail enough to sustain a fracture, whereas those with severe impairment may be protected by their restricted mobility.

Our correlation analyses revealed strong and significant relationships between ECOG, ADL, and mFI-5 fragility scores, demonstrating that these parameters clinically validate each other. Specifically, the strongest correlation between the ADL score and the presence of fracture and the approximately tenfold increase in fracture risk with rising mFI-5 scores demonstrate that functional loss predisposes to fracture through fragility. The significant positive correlation between a history of falls and fracture development underscores how functional decline, when coupled with physical trauma, precipitates fracture risk in the older adult population. Consequently, the pronounced risk of fracture in patients with mild-to-moderate functional decline highlights this subgroup as a high-risk cohort necessitating intensive clinical surveillance. Our findings are in full alignment with the results reported by Edwards et al. (2018), who demonstrated that frailty significantly increases the risk of fracture and that falls in the preceding six months show a trend toward significance with an increased fracture risk in older patients with cancer [6].

The Kaplan–Meier analyses further strengthen our findings by demonstrating that geriatric syndromes not only increase fracture risk but also significantly shorten the time to fracture occurrence. Functional impairment, poor performance status, frailty, malnutrition, and high anticholinergic burden were all associated with earlier fracture events, highlighting the dynamic and time-dependent nature of fracture risk in older adults with bone metastases.

These findings suggest that fractures in this population are not merely cumulative end-stage events but may occur relatively early following the diagnosis of bone metastases, particularly among patients with compromised functional and geriatric profiles. The pronounced reduction in fracture-free survival among patients with post-study falls emphasizes the pivotal role of fall prevention strategies as an integral component of fracture risk mitigation.

Importantly, the observation that patients with moderate functional impairment and frailty experienced earlier fractures than those with severe dependency supports the hypothesis that partial mobility, when combined with skeletal fragility, may expose patients to greater mechanical stress and fall-related trauma. This temporal perspective provided by Kaplan–Meier analysis complements the hazard-based findings and underscores the need for early identification and proactive management of high-risk patients.

Furthermore, as emphasized in a comprehensive review by Bailey et al. (2020), the risk of fracture in metastatic disease is determined by the ratio of daily physical loads to the structural strength of the bone. According to this biomechanical principle, bone tissue, already compromised by metastasis, reaches a point of failure when it can no longer withstand the increased mechanical loads resulting from functional impairments and falls [22]. Within this biomechanical framework, fracture risk reflects the balance between residual bone strength and mobility-related mechanical exposure. Consistent with this principle, in our study, contrary to previous findings [6], baseline BMD was not significantly associated with fractures, highlighting that fracture risk in metastatic bone disease cannot be explained by BMD alone and is more strongly influenced by functional status and geriatric vulnerability.

Nutritional status (MUST) emerged as a significant predictor of fracture risk in our study. Patients at high risk of malnutrition had a fourfold higher risk of fractures. Our findings are consistent with the existing literature, which supports the link between nutritional decline and skeletal compromise. As reported in a cross-sectional study by Zwickl et al. (2021), cancer patients with cachexia exhibit significantly higher levels of bone resorption markers, such as carboxy-terminal telopeptide of collagen, compared to non-cachectic controls. This metabolic shift suggests that malnutrition is not merely a state of general weakness but actively promotes a pro-resorptive environment, thereby increasing skeletal fragility and the likelihood of fractures [23]. Furthermore, the correlation between MUST and ADL scores in our study indicates that malnutrition is closely linked to functional dependency. This relationship suggests that nutritional decline may accelerate sarcopenia, thereby reducing the muscle strength and mechanical support necessary for bone stability.

In our study, a high anticholinergic burden (ACB ≥ 3) was associated with a 4.5-fold increase in fracture risk. Our multivariate analysis further confirmed that a high anticholinergic burden is a potent independent predictor of fracture risk, with patients having an ACB score of ≥ *3* exhibiting a 5.7-fold increased risk compared to those with no anticholinergic load. The positive correlations between ACB, mFI-5 frailty scores, and fracture occurrence suggest that excessive anticholinergic loads exacerbate the frailty syndrome. The significant relationship found between the use of anticholinergic drugs and frailty components in the study by Naharci et al. (2020) supports the results of our study [24]. Conversely, no significant correlation was found between ACB and a history of falls. This discrepancy aligns with the inconsistencies in the literature, where some studies report a strong link between ACB and falls, while others provide inconclusive evidence. For instance, while Markum et al. [25] observed a non-significant increase in recurrent falls among anticholinergic drug users, other studies by Tan et al. [26] and Xu et al. [27] reported a definitive link between high ACB scores and fall risk. The lack of a significant association with falls in our study may be attributed to our relatively small sample size. Although ACB was not statistically associated with recorded falls in our study, anticholinergic burden may contribute to fracture risk through other mechanisms, such as sarcopenia, reduced muscle strength, or gait instability that does not result in a 'fall' event but impairs the ability to dissipate mechanical energy during minor trauma.

Consistent with the findings of Al-Azayzih et al. [28], tramadol use was highly prevalent in our study and significantly associated with fractures. This association may be attributed to tramadol's moderate anticholinergic potency (ACB score of 2), which contributes to cumulative pharmacological vulnerability in older adults. Similarly, prednisolone use emerged as a significant factor in fracture occurrence. Although prednisolone carries a lower anticholinergic load (ACB score of 1) compared to tramadol, its significant association with fractures likely reflects a synergistic effect between its anticholinergic properties and its well-established adverse impact on bone metabolism, such as glucocorticoid-induced bone loss. Overall, our findings demonstrate that a cumulative anticholinergic burden reduces functional independence and exacerbates frailty, thereby predisposing older patients to an increased risk of fractures.

Our results are in strong agreement with the Mirels’ Criteria for Prophylactic Fixation, a widely utilized clinical tool where lesion morphology serves as a primary determinant of fracture risk. In this scoring system, purely osteolytic lesions are assigned the highest risk weight (+ 3 points), followed by mixed lesions containing lytic components (+ 2 points), and lastly, sclerotic lesions (+ 1 point) [29]. This hierarchical risk distribution stems from the pathophysiology of osteolytic destruction, which involves focal bone resorption that severely compromises cortical thickness and trabecular microarchitecture. The nearly fivefold risk increase for lytic lesions and the 3.5-fold increase for mixed lesions observed in our study mirror this structural instability, as the mechanical void created by lytic destruction significantly reduces the bone's load-bearing capacity compared to blastic involvement. This reinforces the clinical necessity of classifying any metastatic involvement with a lytic component as high-risk, consistent with the biomechanical vulnerabilities first identified by Mirels. Beyond mechanical stability, the positive correlation between lesion type and ADL scores indicates that aggressive bone destruction is closely linked to increased functional dependency.

In our study, the presence of lung metastasis emerged as a significant risk factor, associated with a nearly threefold increase in fracture risk. The positive correlations between lung involvement and both MUST and ADL scores suggest that visceral metastasis serves as a marker for advanced systemic disease, characterized by poor nutritional status and functional impairment that predispose patients to skeletal complications. These findings align with the aggressive osteolytic nature of lung cancer, which uniquely disseminates through the arterial flow and the vertebral venous system to the axial skeleton. Once established, metastatic cells trigger a 'vicious cycle' by secreting factors such as PTHrP and RANKL, which activate osteoclasts and severely compromise structural integrity. Furthermore, the anatomical proximity of lung lesions to the thoracic cage and spine inherently increases the risk of mechanical failure in these regions [30].

The present study has several limitations. First, the sample size was relatively limited, which may have constrained the statistical power of the Cox regression analyses. While we identified factors independently associated with fracture risk, the strong intercorrelations among geriatric parameters introduce a potential risk of multicollinearity, necessitating cautious interpretation of these variables as distinct predictors. Regarding the inclusion of post-baseline fall events, these were analyzed as static binary covariates rather than time-dependent variables. Although this approach identifies physical instability as a surrogate for skeletal vulnerability, it lacks the temporal granularity required to establish a definitive causal sequence between specific falls and fractures. Importantly, the study’s primary independent predictors (ECOG and ACB score) were assessed at study entry. This baseline assessment ensures their temporal precedence over the observed outcomes, confirming that the multivariable model’s core findings remain robust despite the concurrent nature of post-baseline fall events. Furthermore, the 6-month follow-up period was primarily designed to capture early skeletal-related events and identify immediate geriatric predictors of fracture risk in a population with advanced cancer. While this timeframe minimizes attrition bias due to the limited life expectancy of older adults with bone metastases, it may not fully reflect the long-term protective effects of antiresorptive agents. Additionally, BMD data were missing for approximately 20% of the population who declined densitometry scans; therefore, the impact of bone density on fracture occurrence may have been masked in our analysis. Despite these constraints, the integration of geriatric parameters provides a realistic reflection of fracture risk in a real-world clinical setting. However, death was treated as a censoring event rather than a competing risk, which may have influenced fracture-free survival estimates.

## Conclusion

In older adult patients with newly diagnosed bone metastases, initiating antiresorptive therapy alone is insufficient as a preventive strategy against fractures. Our study identifies ECOG performance status and high anticholinergic drug burden as the primary independent risk factors for fractures in this population. Furthermore, functional dependency, frailty, malnutrition, and falls were identified as significant contributors to fracture risk. Enhancing current fracture risk scoring systems with comprehensive geriatric assessment components may facilitate more accurate decisions regarding prophylactic surgery and palliative care, thereby potentially contributing to the preservation of the patients' functional quality of life. Furthermore, an approach entailing the multidisciplinary management (geriatricians, clinical pharmacists, dietitians, etc.) of these modifiable risk factors at the time of diagnosis may help mitigate fracture risk. Further longitudinal research with larger, multicenter cohorts and extended observation periods is essential to validate these findings and to more comprehensively evaluate the long-term fracture patterns and the sustained protective efficacy of antiresorptive therapies in this vulnerable population.

## Data Availability

No datasets were generated or analysed during the current study.
